# Navigating pressure and connection: goal orientation, coach-athlete relationships, and burnout among Chinese collegiate athletes—a mixed-methods study

**DOI:** 10.3389/fpsyg.2025.1615274

**Published:** 2025-07-22

**Authors:** Le Wang, Hongye Lian

**Affiliations:** ^1^Department of Physical Education, Zhongyuan University of Technology, Zhengzhou, Henan, China; ^2^Ice and Snow Sports System, Hebei Sport University, Shijiazhuang, Hebei, China

**Keywords:** athlete burnout, goal orientation, coach-athlete relationship, mixed-methods, collegiate athletes, sport psychology

## Abstract

**Introduction:**

Competitive sport often imposes significant physical and psychological stress, making athlete burnout a primary concern. This mixed-methods study investigated the interplay of goal orientation, coach-athlete relationship (CAR) quality, and athlete burnout among Chinese collegiate athletes, considering unique cultural and systemic pressures.

**Methods:**

A sequential explanatory mixed-methods design was employed. The quantitative phase surveyed 346 athletes (M_age_ = 20.6 years) across five Chinese provinces using the TEOSQ (Task and Ego Orientation in Sport Questionnaire), CART-Q (Coach-Athlete Relationship Questionnaire), and RED-A (Reduced Exhaustion Depression and Reduced Accomplishment Scale). Data were analyzed using hierarchical regression. The qualitative phase involved semi-structured interviews with a purposive subsample of 28 diverse athletes, which underwent thematic analysis.

**Results:**

Quantitative findings indicated that task orientation, CAR closeness, and complementarity were significant negative predictors of all burnout dimensions (*p* < 0.05). Ego orientation uniquely predicted higher cynicism (*β* = 0.15, *p* = 0.005). Significant interactions were observed: high closeness was associated with a weaker positive link between ego orientation and cynicism, and high complementarity was associated with an enhanced protective effect of task orientation on reduced accomplishment. CAR commitment did not emerge as a unique predictive factor. Qualitative themes elaborated on these findings, highlighting how performance pressures were associated with goal orientation (e.g., ego orientation linked to cynicism via perceived system barriers) and the importance of specific functional CAR aspects (e.g., complementarity fostering competence, closeness associated with buffering life stress). Interpretations of hardship (specifically “chī kǔ”) also related to task focus and its protective role.

**Discussion:**

The findings underscore that athlete burnout in this context is shaped by motivational orientations and specific relational dynamics within unique cultural and systemic pressures. This suggests that effective interventions should target both individual (e.g., promoting task orientation) and relational factors (e.g., enhancing CAR closeness and complementarity).

## 1 Introduction

Competitive sport demands intense dedication, often imposing significant physical and psychological stress on athletes. While participation offers numerous benefits, athletes are also vulnerable to negative outcomes, with athlete burnout being a primary concern (Eklund and DeFreese, 2015; Gustafsson et al., 2017; Raedeke and Smith, 2001). Defined as a syndrome encompassing emotional exhaustion, sport devaluation (cynicism), and a reduced sense of accomplishment, burnout carries substantial consequences for mental and physical wellbeing, performance, and continued sport participation (Gould et al., 1996; Gustafsson et al., 2011; Smith, 1986). Given evidence suggesting that average burnout symptom levels may be increasing (Madigan et al., 2022), understanding its psychosocial contributors is vital for effective prevention.

Individual motivational factors, such as goal orientations (Nicholls, 1984), play a key role in shaping athletes’ sport experiences. Task orientation, focused on self-referenced mastery and effort, generally is associated with fostering adaptive outcomes like intrinsic motivation, persistence, and lower burnout risk (Martínez-González et al., 2021; Roberts, 2012). Conversely, ego orientation, centered on normative comparison, can be associated with heightened anxiety and maladaptive responses, increasing burnout vulnerability, particularly when superiority is threatened (Duda, 2001; Newton and Duda, 1993).

Equally critical is the social context, especially the coach-athlete relationship (CAR), a central influential factor (Jowett and Cockerill, 2003). The quality of this relationship, often understood through dimensions of closeness, commitment, and complementarity (Jowett, 2007), significantly impacts athlete wellbeing (Mageau and Vallerand, 2003). From a Self-Determination Theory (SDT; Ryan and Deci, 2017) perspective, a high-quality CAR is associated with supporting basic psychological needs for autonomy, competence, and relatedness, contributing to wellbeing and offering protection against burnout (Davis and Jowett, 2014; Felton and Jowett, 2013a, 2013b). Conversely, controlling coaching or poor relationships can be associated with thwarting these needs, increasing burnout risk (Lonsdale et al., 2009; Woods et al., 2022). Thus, both individual motivational factors and the relational context are essential in understanding burnout risk.

Despite research on independent roles, less is understood about the dynamic interplay and interactive influence of goal orientation and CAR on burnout pathways (Isoard-Gautheur et al., 2016). Furthermore, most research originates from Western cultural contexts. A significant gap exists in understanding these dynamics within Chinese collegiate sport, which is characterized by unique systemic pressures (e.g., state-sponsored programs, dual academic-athletic demands) and distinct cultural values, such as collective goals, authority norms, and “chī kǔ” (吃苦)—meaning “eating bitterness” or enduring hardship (Cui et al., 2024; Li et al., 2015; Lenartowicz, 2023). “Chī kǔ” is a deeply ingrained cultural value that emphasizes perseverance through adversity, shaping athletes’ perceptions of effort, challenge, and success in sport (Yang and Jowett, 2013; Si et al., 2015). Prior reliance on predominantly quantitative methods also limits insights into subjective experiences and contextual mechanisms.

Addressing theoretical, contextual, and methodological gaps, this study offers novelty by focusing on goal orientation and CAR interaction within the under-researched Chinese collegiate context, using a mixed-methods design. Guided by SDT, our study had two purposes: (1) to quantitatively examine the unique and interactive contributions of task orientation, ego orientation, and CAR quality (closeness, commitment, complementarity) in predicting athlete burnout among Chinese collegiate athletes; and (2) to utilize qualitative interviews to explore athletes’ lived experiences, providing deeper contextual insights into how these factors influence burnout vulnerability and resilience within their specific environment.

## 2 Literature review

### 2.1 Theoretical framework

This study is grounded in Self-Determination Theory (SDT; Deci and Ryan, 2000; Ryan and Deci, 2017), which posits that individuals possess innate psychological needs for autonomy, competence, and relatedness. SDT provides a robust lens to understand how sport environments, particularly the coach-athlete relationship (CAR), can either support or thwart these needs, thereby impacting athlete motivation and wellbeing (Mageau and Vallerand, 2003; Ryan and Deci, 2020). When needs are frustrated, as often occurs in demanding competitive contexts, psychological ill-being like burnout can emerge (Hodge et al., 2008; Lonsdale et al., 2009; Vansteenkiste and Ryan, 2013).

Achievement Goal Theory (AGT; Nicholls, 1984) complements Self-Determination Theory (SDT) by explaining how task and ego orientations shape athletes’ need satisfaction and burnout risk (Duda, 2001). Task orientation, emphasizing effort and mastery, supports competence and autonomy through self-referenced goals, reducing burnout vulnerability (Nicholls, 1989, 2007). Ego orientation, focused on normative superiority, may frustrate these needs when external validation is unmet, increasing burnout risk, particularly for cynicism. Task-oriented athletes experience relatedness through supportive, growth-focused coach-athlete relationships (CARs), while ego-oriented athletes may perceive relatedness as performance-dependent, straining relational bonds (Duda, 2001). CAR quality, defined by closeness, commitment, and complementarity, mediates these outcomes by fostering or thwarting autonomy, competence, and relatedness (Jowett, 2007; Mageau and Vallerand, 2003). Thus, task orientation buffers burnout by enhancing need satisfaction, while ego orientation heightens vulnerability when needs are frustrated, with CAR quality shaping these dynamics.

In the context of Chinese collegiate sport, the cultural value of “chī kǔ” may theoretically modulate SDT’s basic needs and AGT’s orientations. For instance, competence might be closely tied to enduring hardship effectively, while autonomy could involve embracing strenuous demands as self-chosen growth within a collective framework. These cultural nuances may influence how task and ego orientations interact with CAR quality to affect burnout, highlighting the need for contextual interpretation (Cao et al., 2024; Cui et al., 2024; Lenartowicz, 2023; Si et al., 2015).

### 2.2 Understanding athlete burnout

Athlete burnout, a state characterized by emotional and physical exhaustion, a reduced sense of accomplishment, and sport devaluation (Raedeke and Smith, 2001), is a significant concern in sports psychology. Its notable prevalence across athletic levels (Eklund and DeFreese, 2015; Gustafsson et al., 2017) and potential increase over time (Madigan et al., 2022) necessitate a clear understanding of its complex causes to inform effective interventions. Contributing factors include the chronic stress arising from intense physical and psychological training demands (Smith, 1986), often compounded by individual factors like perfectionism and high personal expectations (Appleton and Hill, 2012; Madigan et al., 2016).

The social environment also plays a critical role. Consistent with Self-Determination Theory (Deci and Ryan, 2000), which highlights fundamental needs for autonomy, competence, and relatedness, environments perceived as controlling or lacking autonomy support can diminish intrinsic motivation and increase burnout risk (Hodge et al., 2008). Similarly, poor coach-athlete relationships and inadequate social support are linked to higher burnout levels (Isoard-Gautheur et al., 2012; Lundkvist et al., 2018), emphasizing the importance of need-supportive contexts.

The consequences of burnout are substantial, affecting both mental and physical health. Athletes experiencing burnout report higher susceptibility to depression and anxiety and lower self-esteem (Gustafsson et al., 2011), alongside potential immune system compromise and impaired performance (Cresswell and Eklund, 2006; Smith, 1986). Furthermore, burnout is a primary predictor of premature sport withdrawal, leading to lost opportunities for athletes and significant disruption for teams and organizations (Cresswell and Eklund, 2005; Gould et al., 1996).

Recent research continues to investigate the multifaceted nature of burnout. A systematic review highlighted the protective roles of autonomy-supportive environments, positive team climates, and self-determined motivation against burnout in team sports, while controlling coaching and socially prescribed perfectionism emerged as risk factors (Woods et al., 2022). Underscoring the centrality of stress, a meta-analysis confirmed a strong positive correlation between athlete stress and burnout, suggesting the potential utility of stress management strategies (Lin et al., 2022). Attention has also turned to potential protective factors and interventions. For instance, psychological resilience has been shown to moderate the relationship between organizational stressors and burnout in young athletes (Wu et al., 2022), while mindfulness training shows promise for reducing burnout and enhancing wellbeing among elite athletes (Zhang et al., 2021). Additionally, research confirms the link between burnout and subsequent health problems, indicating potential negative cycles (Moseid et al., 2023).

### 2.3 Psychosocial influences on burnout

Athlete burnout, from an SDT framework, stems from an interplay of individual and social-environmental factors affecting basic psychological need satisfaction. Key influences include motivational orientations and coach-athlete relationship (CAR) quality. Achievement Goal Theory (AGT; Nicholls, 1984, 1989) identifies two primary dispositional goal orientations: task and ego (Duda and Nicholls, 1992). Task-oriented athletes define success self-referentially, prioritizing effort, learning, and mastery. This supports competence (via personal progress) and fosters autonomous motivation (via intrinsic satisfaction) (Ames, 1992; Duda, 2001). This internal focus builds stress resilience, protecting against burnout (Harris and Smith, 2009). Task-oriented athletes often view demands as growth opportunities, sustaining effort and positive affect (Elliot and Dweck, 1988; Roberts et al., 1997). Empirically, task orientation consistently links to adaptive outcomes, including greater intrinsic motivation, wellbeing, and lower burnout (Martínez-González et al., 2021; Pensgaard and Roberts, 2000; Sorkkila et al., 2018; Van Yperen and Duda, 1999).

In contrast, ego-oriented athletes define competence normatively, seeking superior ability with minimal effort (Nicholls, 1989). Their accomplishment relies on external validation. From an SDT perspective, this carries higher risks for need frustration and burnout. Fragile competence perceptions, reliant on social comparison, lead to anxiety and reduced effort when normative success is threatened (Duda, 2001; Newton and Duda, 1993). This external focus can undermine autonomy, fostering controlled motivation (Ryan and Deci, 2020). When superiority cannot be demonstrated, ego-oriented athletes may experience significant competence need frustration, resulting in inadequacy, enjoyment loss, and maladaptive behaviors like sport withdrawal (Roberts, 2012). This vulnerability can predispose them to higher burnout, including heightened exhaustion and cynicism when normative success proves elusive (Duda, 2001; Sorkkila et al., 2018). However, ego orientation’s role varies, sometimes depending on perceived competence or motivational climate (Duda and Hall, 2001).

Beyond individual motivation, the social environment profoundly shapes athlete experiences. The CAR is arguably the most influential interpersonal dyad in structured sport (Jowett and Cockerill, 2003; Mageau and Vallerand, 2003). Jowett’s (2007) 3 + 1 Cs model frames CAR quality: Closeness (affective connection), Commitment (cognitive attachment), and Complementarity (behavioral interaction). These directly relate to SDT’s basic needs. High Closeness strongly supports relatedness, fostering connection and security (Davis and Jowett, 2014; Felton and Jowett, 2013a, 2013b). Commitment also contributes to relatedness by signaling stability (Jowett and Meek, 2000). Complementarity, through effective interactions and guidance, directly supports athletes’ competence and can foster autonomy via collaboration (Jowett and Poczwardowski, 2007).

Thus, CAR quality critically influences need satisfaction or frustration, directly impacting burnout risk. High-quality relationships (strong 3 Cs) consistently link to positive outcomes: enhanced motivation, wellbeing, and lower burnout (Amorose and Anderson-Butcher, 2007; Davis and Jowett, 2014; Powers et al., 2020; Simons and Bird, 2023). Autonomy-supportive coaches foster higher quality relationships and greater need satisfaction, protecting against burnout (Isoard-Gautheur et al., 2012; Mageau and Vallerand, 2003). Conversely, controlling coaching (e.g., intimidation) thwarts autonomy and relatedness, undermining intrinsic motivation and correlating with higher athlete burnout (Bartholomew et al., 2009; Lonsdale et al., 2009). The coach’s emotional state can also influence athlete wellbeing (Stebbings et al., 2016), highlighting the systemic influence of the relationship.

### 2.4 The interplay between goal orientation and CAR in the burnout process

Despite theoretical expectations, empirical examination of the dynamic relationship between goal orientations and CAR in predicting burnout remains limited, often lacking comprehensive theoretical models or systematic evidence of explanatory mechanisms. While some studies indicate CAR quality can moderate goal orientation effects on wellbeing or burnout (Isoard-Gautheur et al., 2016; Olympiou et al., 2008; Smith et al., 2007)—e.g., potentially buffering ego-oriented consequences or amplifying task-oriented benefits—critical gaps persist. The precise conditions and specific relational dimensions (e.g., closeness, complementarity) influencing different burnout facets (e.g., emotional exhaustion, cynicism) are not systematically delineated. Existing research often presents mixed or incomplete findings (e.g., Fernández-Rio et al., 2017) and largely relies on cross-sectional designs, limiting causal inference (e.g., Isoard-Gautheur et al., 2016; Smith et al., 2007). These collective limitations highlight significant theoretical and empirical gaps in understanding the complex interplay of task and ego orientations with all CAR dimensions across burnout’s multidimensional aspects.

Furthermore, this limited research overwhelmingly focuses on Western cultural contexts, raising questions about cross-cultural applicability. A significant gap exists in understanding these psychosocial dynamics within Chinese collegiate sport, where unique systemic and cultural factors, such as state-sponsored competition and the value of “chī kǔ,” may shape how goal orientations and CAR interact to influence burnout (Cui et al., 2024; Cao et al., 2024; Yang and Jowett, 2013). The mixed-methods design of this study directly addresses the need for insights into subjective experiences and contextual mechanisms underpinning these complex relationships.

### 2.5 The current study

Self-Determination Theory (SDT; Ryan and Deci, 2017) suggests that athlete burnout is related to interactions between goal orientations (task/ego) and coach-athlete relationship (CAR) quality, potentially mediated by the satisfaction of psychological needs (autonomy, competence, relatedness). While goal orientations and CAR independently are associated with burnout (Davis and Jowett, 2014; Lonsdale et al., 2009; Martínez-González et al., 2021; Sorkkila et al., 2018), their interactive effects and underlying mechanisms, such as need satisfaction, remain underexplored (Isoard-Gautheur et al., 2016). Given the unique systemic and cultural factors in Chinese collegiate sport, this study aims to quantitatively examine the contributions of goal orientations and CAR quality to burnout, and qualitatively explore how these factors interact within this specific context (Cui et al., 2024). Prior quantitative approaches also limit insight into athletes’ subjective experiences and contextual influences, potentially hindering effective interventions.

This study addresses theoretical, contextual, and methodological gaps through a sequential explanatory mixed-methods design guided by SDT. For the quantitative phase, we hypothesized: (H1) Task orientation negatively predicts all burnout dimensions (emotional exhaustion, cynicism, reduced accomplishment), reflecting its association with need satisfaction (Martínez-González et al., 2021; Ryan and Deci, 2017; Sorkkila et al., 2018). (H2) Ego orientation positively predicts burnout given its focus on normative success (Duda, 2001; Roberts, 2012). (H3) CAR closeness and complementarity negatively predict burnout, with commitment’s role less certain (Davis and Jowett, 2014; Felton and Jowett, 2013a, 2013b; Mageau and Vallerand, 2003). (RQ1) We explored interaction effects, hypothesizing: (H4a) High closeness is associated with reducing the ego orientation–cynicism link by fostering relatedness, potentially mitigating disengagement in ego-oriented athletes reliant on external validation (Jowett and Cockerill, 2003; Reinboth and Duda, 2006). (H4b) High complementarity is associated with strengthening the negative task orientation–reduced accomplishment link by supporting competence through tailored guidance, thereby reinforcing mastery goals (Jowett and Poczwardowski, 2007; Mageau and Vallerand, 2003).

Therefore, the study aims to: (1) quantitatively examine how task orientation, ego orientation, and CAR quality (closeness, commitment, complementarity) predict burnout dimensions among Chinese collegiate athletes, testing the hypotheses above. (2) Qualitatively explore athletes’ lived experiences to clarify how these factors are associated with interacting within China’s cultural and systemic context, aiming to offer a comprehensive understanding of burnout dynamics.

## 3 Methodology

### 3.1 Participants

The quantitative phase included 346 collegiate athletes (182 males, 164 females; *M*_age = 20.6 years, *SD* = 2.5, range = 16–28) from 12 state-sponsored sports academies and university programs across five Chinese provinces (Guangdong: *n* = 111, 32%; Jiangsu: *n* = 87, 25%; Shandong: *n* = 62, 18%; Zhejiang: *n* = 52, 15%; Fujian: *n* = 34, 10%), selected for socioeconomic and athletic diversity. Eligible participants were enrolled athletes with ≥1 year of formal coaching, ≥4 weekly training sessions or competitions, and registration in provincial or national athlete registries. The sample represented 18 sports: 52% team (*n* = 180; e.g., soccer, basketball) and 48% individual (*n* = 166; e.g., martial arts, swimming). Experience levels were novice (1–3 years: 38%, *n* = 131), intermediate (4–6 years: 45%, *n* = 156), and elite (≥7 years: 17%, *n* = 59). Funding was state-supported for 68% (*n* = 235) and self-funded for 32% (*n* = 111). Weekly training averaged 18.2 h (*SD* = 4.1), with 59% (*n* = 204) competing at provincial or national levels.

Recruitment used digital (e.g., institutional emails, athletic portals, WeChat) and on-site (e.g., training camps, team meetings) methods, coordinated with coaches and administrators, ensuring proportional representation by gender, sport type, and region. A power analysis (G*Power 3.1; Faul et al., 2007) indicated a sample of ~300 achieved 95% power to detect medium effect sizes (*f^2^* = 0.15) in regression with five predictors (*α* = 0.05); the final *n* = 346 met this threshold.

For the qualitative phase, a purposive subsample (*n* = 28; 14 males, 14 females) was selected from the quantitative pool, prioritizing high/low scores (top/bottom 15% for task/ego orientation; top/bottom 10% for burnout) on the Chinese Athlete Burnout Questionnaire, with overlap considered (e.g., high task orientation, low burnout). The subsample ensured diversity in sport type (12 team, 16 individual), region (10 coastal, 8 central, 10 western provinces), and funding status (16 state-funded, 12 self-funded).

Ethical approval was obtained from Hebei Sport University’s Institutional Review Board (ID: HBSU-REC-2023-017, August 12, 2023). Participants received an information sheet detailing study purpose, procedures, risks, benefits, confidentiality, and withdrawal rights. Informed consent was secured, with assent and guardian consent for those under 18. Data were anonymized using unique codes.

### 3.2 Measures

All questionnaires were administered in Mandarin Chinese. Instruments not previously validated in Chinese athletic contexts underwent back-translation by two bilingual experts to ensure linguistic and conceptual equivalence. Confirmatory Factor Analyses (CFAs) in Mplus 8.0 confirmed measurement invariance across gender, sport type, and funding status (*ΔCFI* < 0.01).

#### 3.2.1 Goal orientation

The Task and Ego Orientation in Sport Questionnaire (TEOSQ; Duda, 1989) assessed task (7 items, e.g., “I feel successful when I learn a new skill by trying hard”) and ego orientation (6 items, e.g., “I feel successful when I am the best”) on a 5-point Likert scale (1 = strongly disagree, 5 = strongly agree). The TEOSQ has established validity and reliability (Duda and Nicholls, 1992). In this study, Cronbach’s *α* was 0.84 (task) and 0.88 (ego). CFA supported a two-factor structure: *χ^2^* (64) = 137.60, *p* < 0.001, *χ^2^/df* = 2.15, *CFI* = 0.95, *TLI* = 0.94, *RMSEA* = 0.06, *SRMR* = 0.05, with invariance across gender (*ΔCFI* < 0.01).

#### 3.2.2 Coach-athlete relationship

The Coach-Athlete Relationship Questionnaire (CART-Q; Jowett and Ntoumanis, 2004) measured closeness (4 items, e.g., “I like my coach”), commitment (3 items, e.g., “I am close to my coach”), and complementarity (4 items, e.g., “When coached, I am at ease”) on a 7-point Likert scale (1 = strongly disagree, 7 = strongly agree). The CART-Q has robust psychometric properties (Jowett and Ntoumanis, 2004). Cronbach’s *α* was 0.89 (closeness), 0.85 (commitment), and 0.90 (complementarity). CFA confirmed a three-factor structure: *χ^2^*(41) = 94.30, *p* < 0.001, *χ^2^/df* = 2.30, *CFI* = 0.93, *TLI* = 0.92, *RMSEA* = 0.07, *SRMR* = 0.06, with invariance across sport type (*ΔCFI* < 0.01).

#### 3.2.3 Athlete burnout

The Reduced Exhaustion Depression and Reduced Accomplishment Scale (RED-A; Raedeke and Smith, 2001) was adapted for Chinese athletes via translation, back-translation, and a pilot test (*n* = 25) to ensure item clarity and cultural relevance. The 10-item scale assessed emotional exhaustion (3 items, e.g., “I feel emotionally drained by my sport”), cynicism (3 items, e.g., “I’ve lost enthusiasm for my sport”), and reduced accomplishment (4 items, e.g., “I do not feel competent in my sport”) on an 11-point scale (0 = never, 10 = daily). The scale has strong psychometric properties (Raedeke and Smith, 2001). Cronbach’s *α* was 0.85 (emotional exhaustion), 0.78 (cynicism), and 0.81 (reduced accomplishment). CFA supported a three-factor structure: *χ^2^*(32) = 65.60, *p* < 0.001, *χ^2^/df* = 2.05, *CFI* = 0.96, *TLI* = 0.95, *RMSEA* = 0.06, *SRMR* = 0.05, with invariance across sport type and funding status (*ΔCFI* < 0.01).

#### 3.2.4 Semi-structured interview guide

A semi-structured interview guide explored athletes’ experiences with goal orientation, coach-athlete relationships (CAR), and burnout, building on quantitative findings and existing literature. Open-ended questions addressed goal-setting (e.g., “How do you set training and competition goals?”), coaching dynamics (e.g., “Describe a time your coach’s feedback impacted your motivation”), stress and burnout (e.g., “Have you felt overwhelmed by your sport?”), and coping strategies. Probes (e.g., “Can you provide an example?”) encouraged deeper reflection. The guide was pilot-tested with three collegiate athletes (*n* = 3, excluded from the final sample) to ensure question clarity and effective discussion (Guest et al., 2006).

### 3.3 Procedure

Data were collected from September to November 2023 after obtaining institutional approvals and coach/administrator consent. Participants were recruited via university channels, WeChat, and training camp announcements. Surveys (demographics, TEOSQ, CART-Q, RED-A) were completed online (95%) via a secure platform or on paper (5%), taking 15–20 min. Informed consent was obtained, with anonymity ensured through self-generated codes (e.g., mother’s name initials, birth day). Data were securely stored, with paper surveys manually entered.

Quantitative analysis occurred from December 2023 to January 2024. A purposive subsample (*n* = 28) was contacted for interviews (February–March 2024), conducted face-to-face or via WeChat video, based on participant preference. The first author, fluent in Mandarin and trained in qualitative methods, led all interviews. After reiterating study purpose and obtaining audio-recording consent, interviews followed the guide, lasting 45–60 min, with flexibility for elaboration. Transcriptions were completed verbatim, supplemented by field notes. Thematic saturation was reached after 24 interviews, confirmed by four additional interviews (Guest et al., 2006). Participants received a ¥50 CNY voucher.

### 3.4 Data analysis

Quantitative data were analyzed using IBM SPSS Statistics 28.0. Preliminary analyses checked for missing values, outliers, and assumptions (e.g., normality, linearity, homoscedasticity). Missing data (<3%) were addressed with Full Information Maximum Likelihood (FIML) estimation (Enders, 2010). Descriptive statistics (means, standard deviations, frequencies) and Cronbach’s *α* were calculated for demographic, goal orientation, CAR, and burnout variables.

Pearson correlations (*r*) examined relationships among goal orientation (task, ego), CAR dimensions (closeness, commitment, complementarity), and burnout dimensions (emotional exhaustion, cynicism, reduced accomplishment). Hierarchical regression assessed their contributions to each burnout dimension, using a step-wise approach to evaluate controls, main effects, and interactions. Step 1 included control variables: Athlete Experience Level (1 = novice, 2 = intermediate, 3 = elite), Funding Status (0 = self-funded, 1 = state-funded), and Sport Type (0 = individual, 1 = team), selected for theoretical relevance. Step 2 added goal orientations and CAR dimensions. Step 3 included interactions (Ego Orientation × Closeness, Task Orientation × Complementarity) to test hypotheses (H4a, H4b), as closeness was associated with weaker relationships between ego orientation and cynicism, and complementarity was associated with stronger negative relationships between task orientation and burnout (Jowett and Ntoumanis, 2004; Jowett and Poczwardowski, 2007). Predictors were mean-centered to reduce multicollinearity. Standardized coefficients (*β*) with 95% confidence intervals and *ΔR^2^* values were reported, with significance at *p* < 0.05.

Interview transcripts were analyzed via reflexive thematic analysis (Braun and Clarke, 2006, 2021) in NVivo 12, following six phases: (1) data familiarization through reading and listening, (2) mixed deductive-inductive coding, (3) pattern identification, (4) theme refinement, (5) theme definition and naming, and (6) reporting with illustrative quotes. Two researchers independently coded five interviews (*n* = 5, ~18% of data) to ensure consistency, refining the codebook through discussion. The lead researcher coded remaining data, with checks from the second researcher.

Trustworthiness (Lincoln and Guba, 1985) was enhanced through methodological triangulation (integrating quantitative and qualitative data), peer debriefing, reflexive journaling, thick description, and member checking (*n* = 5). In this sequential explanatory design, integration occurred at multiple points: quantitative findings shaped qualitative sampling and the interview guide, while qualitative themes elaborated and contextualized quantitative results.

## 4 Results

### 4.1 Quantitative results

#### 4.1.1 Descriptive statistics and correlations

Quantitative analyses were conducted using IBM SPSS Statistics 28.0, with significance set at *p* < 0.05. Preliminary checks confirmed minimal missing data (<3%), handled via Full Information Maximum Likelihood (FIML) estimation (Enders, 2010). Diagnostic tests (e.g., variable distributions, scatterplots, residual plots) verified no violations of normality, linearity, multicollinearity (*VIF* < 3.5; tolerance > 0.40), or homoscedasticity, supporting planned correlational and regression analyses. Table 1 presents descriptive statistics (means, standard deviations), Cronbach’s *α*, and Pearson correlations (*r*) for study variables. Scales showed acceptable to excellent reliability. Athletes reported high task orientation, positive CAR perceptions, and moderate burnout levels.

**Table 1:** Descriptive statistics, Cronbach’s alphas, and Pearson correlations among study variables (*N* = 346).

Variable	*M*	*SD*	*α*	1	2	3	4	5	6	7	8
1. Task orientation	4.12	0.78	0.84	—							
2. Ego orientation	3.25	0.91	0.88	0.19***	—						
3. Closeness (CAR)	5.58	1.15	0.89	0.31***	0.08	—					
4. Commitment (CAR)	5.35	1.21	0.85	0.28***	0.11*	0.65***	—				
5. Complementarity (CAR)	5.70	1.09	0.90	0.35***	0.10	0.71***	0.68***	—			
6. Emotional exhaustion	4.65	2.15	0.85	−0.26***	0.16**	−0.42***	−0.38***	−0.45***	—		
7. Cynicism	3.80	1.98	0.78	−0.31***	0.22***	−0.39***	−0.35***	−0.41***	0.62***	—	
8. Reduced accomplishment	4.10	2.05	0.81	−0.38***	0.12*	−0.48***	−0.44***	−0.51***	0.58***	0.65***	—

CAR, Coach-Athlete Relationship. Scale ranges: TEOSQ (1–5), CART-Q (1–7), RED-A (0–10). ^*^p < 0.05, ^**^p < 0.01, ^***^p < 0.001.

Table 1 shows task orientation was negatively associated with all burnout dimensions, suggesting mastery focus is linked to lower burnout. Ego orientation was positively associated with burnout, indicating outcome-focused orientation may be linked to higher burnout risk. CAR dimensions (closeness, commitment, complementarity) were consistently negatively associated with burnout, highlighting their protective role. Burnout dimensions were strongly intercorrelated. Task orientation was positively associated with all CAR dimensions, while ego orientation had weaker, sometimes significant, associations.

#### 4.1.2 Multiple regression analyses predicting burnout dimensions

Three hierarchical regressions examined goal orientation and coach-athlete relationship (CAR) effects on burnout dimensions (Emotional Exhaustion, Cynicism, Reduced Accomplishment). Step 1 incorporated controls: Athlete Experience Level (1 = novice, 2 = intermediate, 3 = elite), Funding Status (0 = self-funded, 1 = state-funded), and Sport Type (0 = individual, 1 = team). Step 2 included Task Orientation, Ego Orientation, Closeness, Commitment, and Complementarity. Step 3 tested interactions (Ego Orientation × Closeness, Task Orientation × Complementarity). Multicollinearity was absent (*VIF* < 4.0). Standardized coefficients (*β*) with 95% confidence intervals from Step 3 were reported. Results are summarized in Table 2.

**Table 2:** Hierarchical multiple regression analyses predicting burnout dimensions (*N* = 346).

Variable	Emotional exhaustion	Cynicism	Reduced accomplishment
	*β* (Step 1)	*β* (Step 2)	*β* (Step 3)
Step 1			
Experience level	0.08	0.03	0.03
Funding status (1 = State)	0.15**	0.12*	0.12*
Sport type (1 = Team)	−0.05	−0.02	−0.02
*R^2^*	0.04**		
*F* for step 1	4.78**		
Step 2			
Task orientation		−0.14**	−0.14**
Ego orientation		0.07	0.07
Closeness (CAR)		−0.21**	−0.21**
Commitment (CAR)		−0.05	−0.05
Complementarity (CAR)		−0.24***	−0.24***
*ΔR^2^*		0.25***	
ΔF for step 2		22.81***	
Step 3			
Task orientation × complementarity			−0.04
Task orientation × closeness			−0.03
Ego orientation × complementarity			0.06
Ego orientation × closeness			−0.05
*ΔR^2^*			0.005
ΔF for step 3			0.52
Final *R^2^*			0.29***
Final *F*			13.91*

*β*, standardized regression coefficient; CAR, Coach-Athlete Relationship. ^*^p < 0.05, ^**^p < 0.01, ^***^p < 0.001.

In Step 1, control variables explained 4% of variance in Emotional Exhaustion [*R^2^* = 0.04, *F* (3, 342) = 4.78, *p* = 0.003]. State-funded status was a significant positive predictor (*β* = 0.15, *p* = 0.006), indicating higher exhaustion was associated with state-funded athletes. In Step 2, including goal orientations and CAR dimensions improved the model [*ΔR^2^* = 0.25, *ΔF* (5, 337) = 22.81, *p* < 0.001]. The final model accounted for 29% of the variance [*R^2^* = 0.29, *F* (8, 337) = 17.15, *p* < 0.001]. Significant negative predictors were Task Orientation (*β* = −0.14, *p* = 0.010), Closeness (*β* = −0.21, *p* = 0.004), and Complementarity (*β* = −0.24, *p* = 0.001). Ego Orientation (*β* = 0.07, *p* = 0.185) and Commitment (*β* = −0.05, *p* = 0.488) were not statistically significant. State-funded status remained statistically significant (*β* = 0.12, *p* = 0.021). In Step 3, interaction terms did not improve the model [*ΔR^2^* = 0.005, *ΔF* (4, 333) = 0.52, *p* = 0.720]. No interaction terms were statistically significant.

For Cynicism, Step 1 explained 6% of the variance [*R^2^* = 0.06, *F* (3, 342) = 7.25, *p* < 0.001]. Higher experience level (*β* = 0.11, *p* = 0.039) and state-funded status (*β* = 0.17, *p* = 0.002) were associated with greater cynicism. Step 2 improved the model [*ΔR^2^* = 0.22, *ΔF* (5, 337) = 20.19, *p* < 0.001], explaining 28% of the variance [*R^2^* = 0.28, *F* (8, 337) = 16.33, *p* < 0.001]. Significant predictors included Task Orientation (*β* = −0.19, *p* < 0.001, negative), Ego Orientation (*β* = 0.15, *p* = 0.005, positive), Closeness (*β* = −0.18, *p* = 0.015, negative), and Complementarity (*β* = −0.20, *p* = 0.008, negative). Commitment (*β* = −0.08, *p* = 0.250) was not statistically significant. State-funded status remained statistically significant (*β* = 0.14, *p* = 0.011). Step 3 interactions improved the model [*ΔR^2^* = 0.02, *ΔF* (4, 333) = 2.05, *p* = 0.042], with Ego Orientation × Closeness significant (*β* = −0.11, *p* = 0.031). At low closeness (1 SD below mean), ego orientation was strongly associated with higher cynicism (*β* = 0.25, *p* < 0.001); at high closeness, this association was weaker (*β* = 0.05, *p* = 0.45).

For Reduced Accomplishment, Step 1 explained 5% of the variance [*R^2^* = 0.05, *F* (3, 342) = 6.01, *p* = 0.001]. Higher experience level (*β* = 0.13, *p* = 0.018) and state-funded status (*β* = 0.14, *p* = 0.010) were associated with greater reduced accomplishment. Step 2 improved the model [*ΔR^2^* = 0.31, *ΔF* (5, 337) = 32.95, *p* < 0.001], explaining 36% of the variance [*R^2^* = 0.36, *F* (8, 337) = 23.74, *p* < 0.001]. Significant negative predictors included Task Orientation (*β* = −0.25, *p* < 0.001), Closeness (*β* = −0.23, *p* = 0.002), and Complementarity (*β* = −0.28, *p* = 0.001). Ego Orientation (*β* = 0.03, *p* = 0.550) and Commitment (*β* = −0.06, *p* = 0.380) were not statistically significant. Experience level remained statistically significant (*β* = 0.09, *p* = 0.045). Step 3 interactions improved the model [*ΔR^2^* = 0.01, *ΔF* (4, 333) = 1.98, *p* = 0.048], with Task Orientation × Complementarity significant (*β* = −0.09, *p* = 0.041). At low complementarity (1 SD below mean), task orientation was associated with lower reduced accomplishment (*β* = −0.15, *p* = 0.02); at high complementarity, this association was stronger (*β* = −0.35, *p* < 0.001). Other interactions were not statistically significant (*ps* > 0.05).

Overall, task orientation, closeness, and complementarity were consistently associated with lower burnout, while ego orientation was associated with higher cynicism. Closeness was associated with a weaker relationship between ego orientation and cynicism, and complementarity was associated with a stronger negative relationship between task orientation and reduced accomplishment. State-funded status and higher experience were associated with higher burnout, reflecting China’s competitive system. These findings highlight how motivation and relational dynamics are associated with burnout.

### 4.2 Qualitative results

The qualitative phase explored the lived experiences behind the quantitative patterns, providing richer context to the interplay of goal orientations, coach-athlete relationships (CAR), and burnout. Thematic analysis of semi-structured interviews with 28 diverse athletes yielded three primary themes: (1) responses to performance pressure: links to goal orientation and burnout; (2) functional aspects of the coach-athlete relationship and burnout; and (3) meaning-making around training hardship (“Chī Kǔ”): links to task orientation and burnout. These themes elaborate on how motivational orientations and relational dynamics manifest within the specific socio-cultural and systemic pressures of Chinese collegiate sport, influencing athlete vulnerability or resilience to burnout. Table 3 summarizes these themes and their sub-themes, offering a clear overview of the qualitative insights.

**Table 3:** Summary of qualitative themes and sub-themes from athlete interviews.

Theme	Sub-theme	Description
1. Responses to performance pressure: links to goal orientation and burnout	1a. Pressure, ego orientation, and conditional self-worth	Athletes high in ego orientation, especially state-funded ones, tied self-worth to performance outcomes, increasing anxiety and burnout when expectations were not met.
	1b. Ego orientation, perceived systemic issues, and cynicism	Ego orientation linked to cynicism when athletes saw the system as unfair (e.g., selection biases), leading to detachment from sport.
	1c. Task orientation as a buffer against performance pressure	Task-oriented athletes focused on effort and skill growth, which protected them from external pressures and lowered burnout risk.
2. Functional aspects of the coach-athlete relationship and burnout	2a. Role of complementarity: effective guidance and athlete competence	Coaches giving specific, process-focused feedback boosted athlete competence, reducing feelings of low accomplishment.
	2b. Role of closeness: acknowledging athlete life stressors	Coaches who showed empathy for life stressors (e.g., academics) built trust, easing emotional exhaustion.
	2c. Commitment: distinction between relational loyalty and functionality	Commitment based on loyalty did not always reduce burnout if the relationship lacked practical or emotional support.
3. Meaning-making around training hardship (“Chī Kǔ”): links to task orientation and burnout	3a. Task orientation and viewing hardship as skill investment	Task-oriented athletes saw “chī kǔ” as valuable for skill-building, boosting motivation and competence.
	3b. Perceived futility of effort and increased burnout risk	When “chī kǔ” felt pointless or unsupported, it heightened exhaustion and cynicism.
	3c. Athlete experience and evolving interpretations of hardship	Experienced athletes viewed hardship differently—some adapted strategically, others felt growing strain over time.

### 4.3 Theme 1: responses to performance pressure: links to goal orientation and burnout

This theme captures how athletes navigated the intense performance expectations prevalent in the Chinese collegiate sports system, often encapsulated by the cultural encouragement to strive relentlessly (“Jiāyóu”). Athletes’ responses to this pressure varied significantly based on their goal orientation, creating distinct pathways toward or away from burnout, particularly for state-funded athletes.

*Sub-theme 1a: pressure, ego orientation, and conditional self-worth:* for athletes high in ego orientation, especially those state-funded, external pressure often linked self-worth directly to performance outcomes. This aligns with quantitative findings associating funding status and ego orientation with higher burnout facets. Failure became more than a setback, impacting core self-value and amplifying anxiety. As Chen Ming (Male, Team Sport, High Ego, High Burnout, State-Funded) described:

*“Every competition feels like a judgment. It’s not just about playing well; it’s about proving the scholarship. If we lose. it feels like you failed everyone. The pressure is not just to win, it’s to be seen as worthy. It’s exhausting living under that microscope.”* This direct quote describes the athletes’ lived experience of conditional self-worth and amplified anxiety under performance pressure.

*Sub-theme 1b: ego orientation, perceived systemic issues, and cynicism:* the positive quantitative link between ego orientation and cynicism was reflected in narratives where athletes developed cynicism, seemingly as a response to frustrated ambitions within a system perceived as sometimes unfair (e.g., selection, resource allocation). Wang Li (Female, Individual Sport, High Ego, High Cynicism) explained:

*“Honestly? Sometimes I think, ‘What’s the point?’ You push and push. but then someone with connections gets the national spot. You start to just… go through the motions. Caring too much only leads to disappointment.”* This narrative illustrates the development of detachment and devaluation of sport.

*Sub-theme 1c: task orientation as a buffer against performance pressure:* in contrast, athletes high in task orientation were better able to manage external pressures by focusing internally on controllable factors like effort and skill improvement, supporting the quantitative finding that task orientation negatively predicted burnout. They interpreted performance demands through a lens of personal development. Zhang Wei (Male, Individual Sport, High Task, Low Burnout) articulated this:

*“Yes, the pressure is always there. But I feel most successful when I nail a technique. or when I execute my race plan perfectly. That feeling of progress, that’s what keeps me going. That’s what I control.”* This internal focus highlights a coping mechanism employed by task-oriented athletes.

### 4.4 Theme 2: functional aspects of the coach-athlete relationship and burnout

This theme moves beyond general notions of support to examine the specific functional qualities of the coach-athlete relationship that appeared protective against burnout. It highlights *how* Closeness and Complementarity, identified as strong predictors quantitatively, operated in practice, and clarifies the role of Commitment.

*Sub-theme 2a: role of complementarity: effective guidance and athlete competence:* the quantitative link between Complementarity and lower Reduced Accomplishment was explained by athletes describing coaches who offered specific, individualized, and process-focused technical feedback. This cooperative dynamic fostered feelings of competence and progress. Liu Jia (Female, Team Sport, High Task, Low Burnout, High Complementarity) stated:

*“My coach does not just say ‘good job.’ He’ll point out exactly what I did well. And when I struggle, he breaks it down technically. I feel like he understands how I learn and perform. It makes me feel capable.”* This quote exemplifies how tailored guidance relates to athletes’ perceived competence.

*Sub-theme 2b: role of closeness: acknowledging athlete life stressors:* the protective effect of Closeness against Emotional Exhaustion seemed related to coaches demonstrating awareness and empathy for athletes’ broader life stressors (e.g., academic, family). This built trust and helped athletes manage their overall emotional load. Li Na (Female, Individual Sport, Low Burnout, High Closeness) shared:

*“Last semester, I was overwhelmed with exams. My coach noticed. helped me talk to my professor, adjusted my training intensity. checked in on how I was coping. Knowing he cared about me as a person, not just an athlete, made a huge difference.”* This narrative illustrates how coaches’ care for the athlete as a person facilitates emotional coping.

*Sub-theme 2c: commitment: distinction between relational loyalty and functionality:* interviews helped clarify why Commitment was a less potent unique predictor of burnout than Closeness or Complementarity. Some athletes expressed high commitment (loyalty) based on relationship length or respect for authority, even if the relationship lacked optimal functional or emotional connection. One athlete (Male, Team Sport, High Commitment, Moderate Burnout) commented:

*“I’ve been with my coach for six years; I respect him immensely and I am committed. But sometimes. I do not feel he really understands the pressure. His feedback is very old-school. I stay because of loyalty, but the training can be draining.”* This quote highlights a distinction between relational loyalty and a relationship’s active burnout-buffering quality.

### 4.5 Theme 3: meaning-making around training hardship (“Chī Kǔ”): links to task orientation and burnout

This theme focuses on how athletes interpreted the culturally valued concept of “chī kǔ” (enduring hardship), particularly relevant given high training demands. How athletes made sense of this hardship was closely linked to their goal orientation and coaching quality, influencing its connection to burnout.

*Sub-theme 3a: task orientation and viewing hardship as skill investment*: task-oriented athletes often framed intense training (“chī kǔ”) as a meaningful and necessary part of skill development, aligning with the negative Task Orientation - > Reduced Accomplishment link. The hardship served a purpose connected to mastery. Zhao Jie (Male, Individual Sport, High Task, Low Burnout) explained:

*“Of course, the training is hard. But when you see that hardship directly translating into better technique. it feels worth it. It’s like forging steel. The ‘bitterness’ is part of the process.”* This framing of hardship illustrates how it can contribute to motivation and perceived competence.

*Sub-theme 3b: perceived futility of effort and increased burnout risk*: burnout symptoms were more pronounced when intense effort felt disconnected from meaningful progress, or when hardship (“chī kǔ”) seemed imposed without clear rationale or adequate coaching support (low CAR). Sun Yue (Female, Team Sport, High Exhaustion, High Cynicism) lamented:

*“We train for hours, drills that feel pointless. The coach just yells ‘Chī kǔ! More effort!’ but does not explain why. You push through the pain, but you feel like a machine. You lose faith that the effort means anything.”* This narrative demonstrates how perceived lack of purpose or support during demanding training can lead to burnout symptoms.

*Sub-theme 3c: athlete experience and evolving interpretations of hardship*: narratives from experienced athletes suggested their interpretation of hardship could evolve, adding nuance to the quantitative finding linking experience to slightly higher burnout. Some learned to manage demands more strategically, while others felt the cumulative impact of years of hardship. An experienced athlete (Female, Individual Sport, Elite, Moderate Burnout) reflected:

*“When I was younger, it was all about winning, pushing through any pain. Now. you realize enduring hardship smartly is key. But the years of intense training… they do leave a mark.”* This reflection highlights the dual role of experience, involving both adaptation and accumulated strain.

In conclusion, the qualitative findings richly contextualize the quantitative results, revealing how goal orientations and coach-athlete relationships influence burnout within Chinese collegiate sports’ high-pressure context. Athletes interpret and experience the cultural emphasis on performance and enduring hardship differently based on their motivational focus (task vs. ego orientation) and coaching relationship quality (complementarity and closeness).

## 5 Discussion

This mixed-methods study examined how goal orientations and coach-athlete relationship (CAR) quality were connected to burnout among Chinese collegiate athletes, guided by Self-Determination Theory (SDT; Ryan and Deci, 2017). Quantitative findings showed task orientation, CAR closeness, and complementarity were negatively linked to all burnout dimensions, while ego orientation was positively linked to cynicism. Commitment did not uniquely predict burnout in multivariate models. Qualitative data enriched these findings by highlighting cultural and systemic influences, such as “chī kǔ,” on athletes’ burnout experiences.

Task orientation consistently predicted lower burnout, aligning with research emphasizing self-referenced success through effort and mastery (Martínez-González et al., 2021; Sorkkila et al., 2018; Van Yperen and Duda, 1999). It is related to supporting competence and autonomy via volitional effort (Ames, 1992; Duda, 2001). Qualitative data indicated task-oriented athletes prioritized skill improvement, gaining intrinsic satisfaction that helped mitigate external pressures. Crucially, they framed “chī kǔ” (enduring hardship) as an essential, purposeful skill investment directly aligned with mastery goals. This cultural understanding facilitated the integration of strenuous training into personal growth, leading to enhanced perceived accomplishment and making intense effort inherently meaningful (Harris and Smith, 2009; Roberts et al., 1997). This proactive “chī kǔ” interpretation functions as a unique cultural mechanism, contributing to strengthening task orientation’s buffering effect on burnout and offering a distinct pathway to wellbeing compared to contexts where hardship is primarily viewed as a negative cost. Promoting task orientation may thus be linked to reducing burnout risk in Chinese collegiate sport by fostering intrinsic motivation.

In contrast, ego orientation uniquely increased cynicism, though not emotional exhaustion or reduced accomplishment (Duda, 2001; Nicholls, 1989). SDT suggests that seeking external validation may weaken competence, with task orientation and CAR quality moderating broader burnout effects (Deci and Ryan, 2000). Qualitative findings showed high-ego athletes developed cynicism when ambitions clashed with systemic barriers, such as selection politics, suggesting defensive disengagement (Duda and Hall, 2001; Roberts, 2012). For them, “chī kǔ” became futile without normative success, transforming endured hardship into disappointment and detachment that fueled cynicism. This indicates that, for ego-oriented athletes, “chī kǔ” requires external validation to serve its cultural purpose; without it, resentment and disengagement emerge (Yang and Jowett, 2013). Examples from Chen Ming and Wang Li highlighted how performance pressures and unmet ego needs tied to systemic barriers deepened exhaustion and cynicism, contextualizing this pathway.

Coach-athlete relationship (CAR) quality, aligned with SDT’s focus on social environments, was strongly linked to burnout (Deci and Ryan, 2000). Closeness and complementarity negatively predicted all burnout dimensions, highlighting their protective roles (Davis and Jowett, 2014; Lonsdale et al., 2009; Simons and Bird, 2023). Closeness, reflecting trust and emotional bonds (Jowett and Ntoumanis, 2004), was found to support relatedness, contributing to reduced emotional exhaustion through care beyond sport (Felton and Jowett, 2013a, 2013b). Complementarity, marked by effective interactions (Jowett and Poczwardowski, 2007), appeared to enhance competence and autonomy via tailored guidance, leading to decreased reduced accomplishment (Isoard-Gautheur et al., 2012; Mageau and Vallerand, 2003). Unlike controlling coaching, which is often associated with increased burnout risk (Bartholomew et al., 2009), these dynamics were critical. Qualitative findings, such as Liu Jia’s account of guidance helping to boost accomplishment and Li Na’s experience of empathy helping to ease emotional load, supported these effects. Commitment, despite negative bivariate correlations with burnout, was not a unique predictor in multivariate models (Jowett and Meek, 2000). Qualitative data clarified that commitment often stemmed from loyalty or respect for authority, even when lacking strong closeness or complementarity. This suggests that commitment alone may not adequately address burnout without fulfilling psychological needs through emotional and interactive support (Deci and Ryan, 2000).

The findings highlight the interplay between goal orientation and CAR quality in shaping burnout, as supported by SDT. Qualitative data and prior research confirm these interactive dynamics are key to understanding burnout (Isoard-Gautheur et al., 2016; Olympiou et al., 2008; Smith et al., 2007). High-quality CARs can be linked to buffering ego orientation’s burnout risks or to enhancing task orientation’s protective effects. For instance, strong complementarity may be associated with reducing ego orientation’s link to reduced accomplishment by fostering competence through consistent feedback, while poor CARs may be associated with heightening ego-related stress, potentially increasing burnout. Task-oriented athletes, valuing collaboration, likely tend to benefit from coaches offering supportive guidance (high complementarity), whereas ego-oriented athletes may prioritize coaches who validate superiority, potentially undervaluing cooperative interactions (Reinboth and Duda, 2006). Within SDT, resilience to burnout is suggested to be strengthened when task orientation aligns with a supportive CAR (high closeness and complementarity), thereby satisfying competence, autonomy, and relatedness needs (Lonsdale et al., 2009; Vansteenkiste and Ryan, 2013). Conversely, high ego orientation in a need-thwarting context (e.g., controlling coaching, low closeness) is related to risking need frustration, potentially leading to exhaustion, cynicism, and reduced accomplishment. These findings emphasize the combined role of motivation and relational dynamics in burnout prevention.

Our quantitative analysis of interaction effects revealed important nuances in how goal orientations and CAR quality combine to influence burnout, addressing a key gap. The significant negative interaction between ego orientation and closeness directly supported our hypothesis (H4a): strong emotional bonds with coaches are associated with significantly reduced cynicism in ego-oriented athletes. This aligns with a close relationship fostering relatedness (SDT; Ryan and Deci, 2017), providing a secure interpersonal base that may help ego-oriented athletes reframe competitive setbacks and perceive systemic barriers less as personal threats, thus reducing need frustration that often fuels cynical detachment (Duda, 2001; Lonsdale et al., 2009). Similarly, the significant negative interaction between task orientation and complementarity supported our hypothesis (H4b): effective coaching guidance was related to enhancing task orientation’s protective effect on reduced accomplishment. For task-oriented athletes, complementarity (e.g., clear, individualized, process-focused feedback) directly supports their need for competence (SDT; Deci and Ryan, 2000), validating effort and progress (Nicholls, 1984). This contributes to strengthening intrinsic motivation and enhancing accomplishment, thereby being related to preventing inadequacy (Roberts, 2012). Qualitative data supported these interpretations: closeness was related to mitigating stress, helping athletes cope with setbacks and perceived systemic unfairness. Effective guidance was linked to amplifying mastery benefits, aligning with qualitative findings emphasizing complementarity’s role in fostering competence and making training meaningful. Without such coaching, task-oriented athletes may perceive efforts as futile, potentially increasing burnout risk. These empirical findings collectively underscore the importance of the dynamic interplay between individual motivation and relational context, as strongly suggested by SDT. Our results provide evidence for these interactive dynamics.

Chinese collegiate sport’s unique cultural context profoundly shapes these findings. In a society valuing collectivism and hierarchical authority (Cui et al., 2024; Si et al., 2015), the coach-athlete relationship often reflects paternalistic care and authoritative mentorship. This dynamic fundamentally differs from the more egalitarian closeness typically emphasized in Western frameworks (Yang and Jowett, 2013; Li et al., 2015; Lenartowicz, 2023). For instance, Li Na′s narrative (Theme 2b) described closeness as a coach providing guidance and support within a hierarchical structure, aligning with traditional cultural norms. This culturally specific closeness effectively buffers burnout by meeting athlete expectations of relatedness through respected mentorship. Similarly, complementarity, reflecting effective and cooperative coach-athlete interactions, is observed to thrive when it successfully blends traditional Chinese instructional methods with more collaborative guidance, allowing for knowledge transfer within a respected power dynamic and fostering a culturally congruent environment for skill development (Yang and Jowett, 2013; Jin et al., 2022).

Crucially, “chī kǔ” (enduring hardship) serves as a central lens through which athletes interpret training demands and their vulnerability to burnout (Theme 3). Qualitative findings vividly demonstrated its interaction with goal orientations: task-oriented athletes actively framed “chī kǔ” as a meaningful, necessary component of personal growth and skill investment. This culturally resonant interpretation of effort was linked to enhanced competence and intrinsic motivation, thereby mitigating burnout. This proactive embrace of hardship as a path to mastery highlights a distinct pathway to wellbeing in a context where enduring adversity is a celebrated trait, a dynamic often explored in Chinese sport psychology research emphasizing “adversity training models” (e.g., Zhang et al., 2021). Conversely, for ego-oriented athletes, the intense effort embodying “chī kǔ” could become a source of frustration and cynicism if it did not yield external recognition or superior performance, especially amidst perceived systemic barriers (e.g., selection politics). The perceived futility of their “chī kǔ” in achieving norm-referenced goals was linked to amplified disappointment and detachment, demonstrating how the meaning-making around this cultural concept may either protect or exacerbate burnout depending on an athlete’s motivational orientation and the supportive context. Collectivism also is related to amplifying state-sponsored pressures, contributing to higher burnout among state-funded athletes who carry collective expectations (Cui et al., 2024; Si et al., 2015). These interwoven cultural factors, illuminated by our mixed-methods approach, highlight why direct application of Western-centric psychological models without cultural adaptation is associated with risking ineffective interventions in diverse athletic settings.

## 6 Limitations and future directions

This study offers several strengths for understanding athlete burnout in the Chinese collegiate context. Its sequential explanatory mixed-methods design is a key advantage, providing statistical identification of relationships alongside in-depth qualitative exploration of mechanisms and lived experiences within this under-researched population. Furthermore, using established psychometric measures with demonstrated reliability, combined with rigorous qualitative analysis strategies (e.g., multiple researchers, peer debriefing, member checking), enhances confidence in the findings’ validity and trustworthiness.

However, certain limitations warrant consideration. First, cross-sectional quantitative data preclude definitive inferences about causality or influence direction. Longitudinal research is necessary to determine if poor relationships prospectively predict burnout, or if increasing burnout levels erode athletes’ relationship perceptions. Second, reliance on self-report measures introduces potential common method variance and susceptibility to social desirability or recall biases. Social desirability bias is particularly salient in cultural contexts with high respect for authority, such as China, where athletes may report more socially desirable responses regarding coaches or the sport system, potentially attenuating observed effects. Qualitative data integration provides some triangulation against this bias.

Third, our sample’s geographical representation and overall representativeness are notable limitations. Participants were recruited from institutions across five provinces, primarily among China’s most economically developed regions. Consequently, findings may not fully generalize to collegiate athletes in inland or western provinces, where sport development, institutional structures, and cultural nuances might differ. This sampling approach may thus underestimate stress levels experienced by athletes from less developed central and western regions. For instance, athletes in such regions might face different resource constraints, institutional pressures, or interpretations of “chī kǔ,” potentially altering burnout prevalence or relationships. Furthermore, the specific distribution of athlete experience levels (38% novice, 45% intermediate, 17% elite) might influence observed burnout levels. Elite athletes, though fewer, may experience heightened pressures or different coping mechanisms. Therefore, while our sample offers valuable insights into collegiate athletes in key Chinese provinces, it may not fully represent the diverse athletic landscape across all regions or competitive tiers in Mainland China, limiting broader generalizability.

Finally, our regression models did not account for all potential confounders. Variables like perfectionism (Appleton and Hill, 2012; Madigan et al., 2016) and academic stress are established burnout predictors not included in our analyses. Their omission may have influenced observed effect sizes. For instance, maladaptive perfectionism might amplify ego orientation’s link to cynicism by increasing self-criticism when normative goals are unmet. Conversely, adaptive perfectionism could strengthen task orientation’s protective effects. Similarly, high academic stress, prevalent for Chinese collegiate athletes, could exacerbate emotional exhaustion independently or interact with goal orientation/CAR quality, intensifying burnout with fragile ego-orientation or less supportive coaching. Moreover, excluding these variables limits direct comparisons with Western contexts. Some Western research suggests a stronger, more direct link between ego orientation and all burnout dimensions, possibly due to unmeasured perfectionistic traits or distinct academic systems in those populations. While hierarchical regression suited our primary objectives, future research could leverage more robust alternatives like structural equation modeling (SEM) to explore latent relationships and model fit.

A critical limitation is the absence of direct quantitative measures for specific cultural and institutional characteristics (e.g., collectivist norms, state-sponsored training structures). Our study did not statistically test their moderating effects. Thus, while our theoretical framework acknowledges broader contextual influences, we could not operationalize them as quantitative variables or direct moderators. Our mixed-methods approach, however, provided valuable qualitative insights into how the Chinese sociopsychological context manifests in athlete experiences. Themes like “chī kǔ” interpretation, performance pressures linked to state-funded status, and nuanced CAR dynamics (e.g., authority, loyalty) emerged, illustrating specific cultural and institutional influences on motivation, CARs, and burnout. Nonetheless, the lack of quantitative operationalization for these cultural characteristics remains a limitation for statistically evaluating their moderating effects. Future studies are strongly encouraged to develop validated scales for constructs like “chī kǔ” or collectivism within sports, empirically measuring their impact and testing moderating roles in longitudinal designs, paving the way for comprehensive, culturally tailored interventions.

These limitations highlight crucial avenues for future research. Longitudinal studies are essential to track burnout trajectories and reciprocal dynamics between goal orientations, CAR quality, and need satisfaction. This includes further investigating the ego orientation-cynicism link and coach-athlete closeness’s moderating role. Future work could also benefit from multi-source data, incorporating coach perspectives or objective physiological stress markers. To enhance generalizability within China, expanding national recruitment across a wider range of institutions, including central, inland, and western provinces, would be beneficial. Furthermore, research could explicitly explore potential mediators like basic need satisfaction using quantitative mediation models incorporating identified interaction effects. Investigating how specific Chinese cultural factors (e.g., “face” sensitivity, “guanxi” dynamics) might further moderate observed relationships would also be valuable. Finally, experimental or intervention studies based on these findings are warranted. Such studies could test programs enhancing coach complementarity skills (amplifying task orientation’s protective effects) or helping ego-oriented athletes develop adaptive coping strategies for systemic pressures while fostering stronger coach-athlete closeness.

## 7 Implications and conclusion

The findings offer several specific practical implications, primarily for stakeholders aiming to prevent athlete burnout within the Chinese collegiate sport system. These implications extend beyond broad recommendations to propose concrete, actionable strategies for interventions and institutional support. A dual focus is essential, targeting both individual athlete motivation and the coaching environment. To cultivate task orientation and build athlete resilience, particularly given qualitative insights on “chī kǔ” (enduring hardship), concrete strategies include implementing individualized goal-setting workshops focused on personal mastery and effort; providing regular, specific, and process-oriented feedback rather than solely outcome-based evaluations; and designing training drills that allow for progressive skill acquisition.

Coaches are pivotal and require targeted training beyond general positivity. To enhance emotional closeness, programs should train coaches in active listening and empathetic communication, encourage informal check-ins on athlete academic and personal lives, and foster an environment where athletes feel safe to discuss non-sport-related challenges without fear of judgment. To improve functional complementarity, strategies include implementing structured coach education programs on autonomy-supportive coaching behaviors, encouraging coaches to co-create training plans with athletes, and promoting video analysis paired with one-on-one technical debriefs focused on specific skill refinement.

The finding that relationship commitment alone did not uniquely predict lower burnout underscores that interventions must emphasize quality of interaction (closeness and complementarity) rather than solely relationship stability. Furthermore, the unique link between ego orientation and cynicism, buffered by coach-athlete closeness, suggests a need for tailored support for highly ego-involved athletes. Specific interventions could involve developing resilience-building modules for athletes on re-framing failures as learning opportunities, and training coaches to address systemic issues (e.g., selection transparency) openly and fairly. Coaches actively building close relationships remain crucial to mitigate cynicism and prevent sport devaluation. It is crucial to recognize that these interventions should be developed and implemented with careful consideration of the specific cultural norms and values within the Chinese context, and their direct applicability to other contexts should be approached with caution.

In conclusion, this mixed-methods study provides compelling evidence for the interactive roles of goal orientation and coach-athlete relationship quality in predicting burnout among Chinese collegiate athletes, interpreted through the lens of Self-Determination Theory. Task orientation, closeness, and complementarity emerged as key protective factors, likely by fostering satisfaction of basic psychological needs. Ego orientation represented a specific risk factor for cynicism, which was significantly mitigated by coach-athlete closeness. Moreover, task orientation protective effect on reduced accomplishment was enhanced by coach-athlete complementarity. The sequential explanatory mixed-methods design proved invaluable, offering a uniquely profound and contextually grounded understanding of burnout dynamics by providing empirical evidence for complex relationships while simultaneously uncovering the lived experiences and cultural nuances that explain *how* and *why* these patterns manifest in Chinese collegiate sport. In the Chinese collegiate context, promoting athlete wellbeing requires a holistic approach that nurtures adaptive motivation and fosters supportive, functional coach-athlete connections within the specific cultural and systemic realities athletes face.

## Data Availability

The raw data supporting the conclusions of this article will be made available by the authors, without undue reservation.
